# Combinations of stroke neurorehabilitation to facilitate motor recovery: perspectives on Hebbian plasticity and homeostatic metaplasticity

**DOI:** 10.3389/fnhum.2015.00349

**Published:** 2015-06-23

**Authors:** Naoyuki Takeuchi, Shin-Ichi Izumi

**Affiliations:** Department of Physical Medicine and Rehabilitation, Tohoku University Graduate School of MedicineSendai, Japan

**Keywords:** stroke, motor recovery, rehabilitation, Hebbian plasticity, homeostatic metaplasticity

## Abstract

Motor recovery after stroke involves developing new neural connections, acquiring new functions, and compensating for impairments. These processes are related to neural plasticity. Various novel stroke rehabilitation techniques based on basic science and clinical studies of neural plasticity have been developed to aid motor recovery. Current research aims to determine whether using combinations of these techniques can synergistically improve motor recovery. When different stroke neurorehabilitation therapies are combined, the timing of each therapeutic program must be considered to enable optimal neural plasticity. Synchronizing stroke rehabilitation with voluntary neural and/or muscle activity can lead to motor recovery by targeting Hebbian plasticity. This reinforces the neural connections between paretic muscles and the residual motor area. Homeostatic metaplasticity, which stabilizes the activity of neurons and neural circuits, can either augment or reduce the synergic effect depending on the timing of combination therapy and types of neurorehabilitation that are used. Moreover, the possibility that the threshold and degree of induced plasticity can be altered after stroke should be noted. This review focuses on the mechanisms underlying combinations of neurorehabilitation approaches and their future clinical applications. We suggest therapeutic approaches for cortical reorganization and maximal functional gain in patients with stroke, based on the processes of Hebbian plasticity and homeostatic metaplasticity. Few of the possible combinations of stroke neurorehabilitation have been tested experimentally; therefore, further studies are required to determine the appropriate combination for motor recovery.

## Introduction

Advances in non-invasive brain imaging technologies have increased our understanding of neural plasticity, which induces the functional and structural changes in the central nervous system after stroke and rehabilitation (Lindenberg et al., 2010a; Rehme et al., 2011; Westlake et al., 2012; De Vico Fallani et al., 2013; Grefkes and Ward, 2014; Liuzzi et al., 2014; Stinear and Byblow, 2014; Schulz et al., 2015). Neural plasticity is the ability of the brain to develop new neuronal connections, acquire new functions, and compensate for impairments (Murphy and Corbett, 2009, Dimyan and Cohen, 2011, Takeuchi and Izumi, 2012a). These processes are crucial for motor recovery after stroke. Although various neurorehabilitation strategies are emerging to enhance beneficial plasticity and improve motor recovery after stroke, these interventions show large inter-individual variations in efficacy, due to the heterogeneous mechanisms underlying motor recovery across patients (Langhorne et al., 2011; Pollock et al., 2014; Veerbeek et al., 2014). Therefore, investigations are underway to determine if employing combinations of these techniques can maximize motor recovery by strengthening neural plasticity. Combining protocols that are based on different mechanisms, rather than using each therapy alone, is expected to complement and augment the effects of each therapy to more stably and synergistically facilitate motor recovery.

However, when stroke neurorehabilitation therapies are combined, Hebbian plasticity and homeostatic metaplasticity, as well as ceiling effects, must be taken into account. New or reinforced neural connections induced by Hebbian plasticity is proposed to target plasticity primarily between paretic muscles and residual cortical representations, providing a more focused rehabilitation strategy (Edwardson et al., 2013). The time window for long-term potentiation (LTP)-like plasticity between the cortex and periphery is very narrow and a delay in brain stimulation following movement onset might conversely induce long-term depression (LTD)-like plasticity (Thabit et al., 2010; Massie et al., 2015). Facilitatory priming followed by other facilitatory strategies may diminish the aftereffects of the latter by triggering homeostatic metaplasticity that maintains neural network activity (Abraham, 2008; Jung and Ziemann, 2009; Fricke et al., 2011).

In this review, we first overview the principles of Hebbian plasticity and homeostatic metaplasticity that underlie these synergic effects. We then discuss the different combinations of neurorehabilitation therapy in relation to distinct or simultaneous timing. The concept of priming helps us to understand the interaction between neurorehabilitation therapies may be difficult to perform simultaneously; however, inappropriate timing between interventions that may cause their synergic effect to disappear due to homeostatic metaplasticity. Next, we discuss how simultaneous central or peripheral stimulation contingent on voluntary neural or muscle activity can induce activity-dependent plasticity. We then describe the development of technology that can provide real-time brain activity monitoring that will assist in identifying the narrow time window available to induce activity-dependent plasticity for motor recovery after stroke. Lastly, we discuss the future of combined neurorehabilitation to facilitate the synergy of these processes. Combinations of stroke motor neurorehabilitation techniques are currently being applied clinically; therefore, for future research, it is important to discuss current practices and highlight the gaps in our knowledge.

## Hebbian plasticity and homeostatic metaplasticity

Neural plasticity is tightly regulated and can be significantly affected by the timing of when stroke neurorehabilitation therapies are combined. In this section, we overview activity-dependent stimulation targeting Hebbian plasticity and homeostatic metaplasticity, which may influence the timing of therapy with respect to the effectiveness of combined stroke neurorehabilitation.

### Activity-dependent stimulation targeting Hebbian plasticity

There is much evidence from basic science about the important roles of activity-dependent plasticity after motor learning (Classen et al., 1998; Butefisch et al., 2000; Muellbacher et al., 2001). In the human motor system, it has been well described that activity-dependent plasticity can be artificially induced by brain stimulation in a manner contingent on strict temporal relationships in central or peripheral voluntary activity. This method is based on Hebbian plasticity, in which synaptic plasticity is strengthened when presynaptic and postsynaptic neurons are coincidentally active (Hebb, 1949). In humans, paired associative stimulation (PAS) is the most studied methodology for inducing activity-dependent cortical stimulation (Stefan et al., 2000; Muller-Dahlhaus et al., 2010). PAS is carried out using low-frequency pairing median nerve stimulation at the wrist with delayed transcranial magnetic stimulation (TMS) to the region of the contralateral motor cortex (M1) that represents the thumb (Stefan et al., 2000). This LTP/LTD-like plasticity is dependent on the timing between median nerve stimulation and TMS. LTP-like plasticity is induced when median nerve stimulation precedes TMS by 25 or 2 ms after the individual N20 latency of the median nerve somatosensory evoked potential, whereas LTD-like plasticity is induced using an intersimulus interval of 10 or 5 ms before the N20 latency (Wolters et al., 2003; Ziemann et al., 2004; Muller et al., 2007).

In addition to peripheral sensory stimulation, other studies have revealed that activity-dependent cortical stimulation by TMS over M1 synchronizes with voluntary muscle movement. Butefisch et al. studied whether TMS over the M1 synchronously applied with motor training could enhance the encoding of motor memory (Butefisch et al., 2004). Subjects practiced 1 Hz 30-min brisk thumb voluntary movements in the opposite direction evoked by TMS over the M1 region that represents the thumb movement. A subthreshold TMS triggered by the muscle activity was applied at 0.1 Hz to the contralateral or ipsilateral M1 in synchrony with 1 out of every 10 voluntary thumb movements. This study showed that movement-dependent encoding of a motor memory for a trained direction can be enhanced by synchronous TMS at the contralateral M1 and reduced by TMS at the homologous ipsilateral M1. Thabit et al. reported that TMS over M1 paired with 0.2 Hz 20-min thumb voluntary movement can induce changes in the corticospinal excitability and motor behavior (Thabit et al., 2010). When TMS was delivered 50 ms before movement reaction time, the cortical excitability of the target muscle increased for up to 15 min after stimulation. This also shortened the target muscle reaction time. However, when TMS was delivered 100 ms after the reaction time, cortical excitability was decreased. Massie et al. investigated the use of synchronous TMS over M1 during robotic reaching training in the opposite direction evoked by TMS (Massie et al., 2015). TMS was synchronously applied during alternate reaching tasks that consisted of three blocks of 160 trials. When TMS was delivered approximately 150 ms before the reaction time, M1 cortical excitability representing muscles involved in the reaching movement increased. However, when TMS was delivered at the time of movement onset, as triggered by muscle activity, cortical excitability decreased. Interestingly, motor performance significantly improved following training but did not differ between conditions. Thus, there is a very narrow time window available between cortical and peripheral activity to induce LTP-like and LTD-like plasticity.

### Homeostatic metaplasticity

Synaptic plasticity regulated by LTP and LTD can become excessive through activation of a positive feedback loop (Abraham, 2008; Murphy and Corbett, 2009). To counteract this positive feedback loop and maintain a physiological range of synaptic plasticity, negative-feedback is necessary (Murphy and Corbett, 2009). Homeostatic metaplasticity is a form of synaptic plasticity that modifies and maintains the stability of neuron and neuronal network activity within the physiological range (Abraham, 2008; Turrigiano, 2008; Murphy and Corbett, 2009). A conceptual basis of homeostatic metaplasticity is provided by the Bienenstock–Cooper–Munro (BCM) theory: the threshold for induction of LTP or LTD is dynamically adjusted according to the history of activation (Bienenstock et al., 1982). An increase in the synaptic modification threshold by recent high-level activity will favor the induction of LTD over LTP. Conversely, a reduction in the threshold due to low-level activity will favor the induction of LTP over LTD (Bienenstock et al., 1982).

In the human motor system, many studies have reported interactions between interventions *via* homeostatic metaplasticity by combining two non-invasive brain stimulation (NIBS) techniques, which can change cortical excitability (Iyer et al., 2003; Lang et al., 2004; Hamada et al., 2008; Fricke et al., 2011; Murakami et al., 2012). However, it is important for the continuous improvement of motor function after stroke that the combination of techniques for neurorehabilitation are based on motor learning, rather than NIBS intervention alone. Therefore, in this section, we mainly discuss homeostatic plasticity elicited when combining NIBS with motor learning.

Jung and Ziemann evaluated the correlation between LTP/LTD-like plasticity induced by PAS and motor learning in healthy subjects (Jung and Ziemann, 2009). They reported that this combination of facilitation techniques is influenced by homeostatic metaplasticity if there is an extended period of time between interventions. Motor training immediately following LTD-like plasticity enhanced motor learning according to homeostatic interactions. In addition, motor training immediately following LTP-like plasticity also enhanced motor learning, although to a lesser extent. However, if motor training was undertaken 90 min after PAS, LTD-like plasticity facilitated motor learning, whereas LTP-like plasticity depressed motor learning. Therefore, subsequent facilitation techniques occurring with a long time delay after the first facilitation program are easily influenced by homeostatic interactions, whereas a synergic effect of combined facilitatory approaches without a delay is expected because homeostatic interactions are avoided. Animal studies showing that non-saturated LTP facilitated subsequent learning may provide some explanation for the non-homeostatic interactions between LTP-like plasticity and immediately subsequent motor learning (Berger, 1984; Jeffery and Morris, 1993). These results indicate that neurorehabilitation based on motor learning immediately followed by a facilitation technique might avoid a reduction in the synergic effect due to homeostatic metaplasticity. This is consistent with another study showing that priming with excitatory intermittent theta burst stimulation (iTBS), with an interval of 10 min between iTBS and motor training, enhanced the subsequent motor learning of ballistic thumb movements (Teo et al., 2011). However, the effect of subsequent motor training may depend on the type of NIBS; Kuo et al. reported that excitatory anodal transcranial direct current stimulation (tDCS) over M1 immediately before a serial reaction time task does not affect implicit motor learning (Kuo et al., 2008). Conversely, Nitsche et al. demonstrated that the application of anodal tDCS during the same task leads to an improvement in implicit motor learning (Nitsche et al., 2003). Stagg et al. have also shown that anodal tDCS improves explicit motor learning when applied during the motor task, but not if it is applied before the task (Stagg et al., 2011). Therefore, homeostatic effects may occur in M1 when excitatory tDCS is applied before motor training that increases excitability in an activity-dependent manner.

On the other hand, simultaneous timing between interventions that are based on similar mechanisms is always not desirable for a synergic effect. Nitsche et al. investigated whether the timing between tDCS and LTP-like plasticity induced by PAS influences homeostatic metaplasticity (Nitsche et al., 2007). They reported that preconditioning M1 with excitatory tDCS enhances the subsequent PAS-induced LTP-like plasticity, whereas inhibitory tDCS reduces subsequent LTP-like plasticity. In contrast, when tDCS and LTP-like plasticity are applied simultaneously, inhibitory tDCS results in a prolonged LTP-like plasticity, whereas excitatory tDCS produces inhibition of LTP-like plasticity. Thus, the effects of homeostatic metaplasticity may differ depending on the type of combined interventions, in addition to their timing.

In next section, we discuss the combination therapies currently used to treat patients with stroke in the context of homeostatic metaplasticity and Hebbian plasticity. However, research findings describing homeostatic metaplasticity and Hebbian plasticity are mainly obtained from young healthy subjects. Moreover, there is considerable evidence that activity-dependent plasticity after motor learning is reduced in older subjects compared to young ones (Sawaki et al., 2003; Zimerman et al., 2013). Furthermore, the neural plasticity induced by NIBS is different between young and older subjects (Ridding and Ziemann, 2010; Zimerman et al., 2013). Age-related neural plasticity changes are known to result from region-specific changes in dendritic morphology, cellular connectivity, Ca^2+^ dysregulation, and gene expression, amongst other factors (Burke and Barnes, 2006). In addition to aging, stroke itself induces structural and functional changes in the brain (Murphy and Corbett, 2009; Starkey and Schwab, 2014). Therefore, the effect of combinatorial interventions in patients with stroke might differ from those predicted from findings in young healthy subjects due to structural and functional changes in aging and/or neural reorganization after stroke (Burke and Barnes, 2006; Murphy and Corbett, 2009; Heise et al., 2013; Starkey and Schwab, 2014).

## Combined neurorehabilititation therapies for motor recovery after stroke

Over the last several decades, many studies have reported various motor learning-based stroke rehabilitation strategies (Langhorne et al., 2009; Johansson, 2012; Pollock et al., 2014; Veerbeek et al., 2014). Constraint-induced movement therapy (CIMT), which combines a rehabilitative training regime for the paretic limb with non-paretic limb restraint, can overcome learned nonuse of the paretic limb and has been shown to improve motor function in patients with stroke. Robotic training can provide repetitive motor training and intensive practice. NIBS and neuromuscular electrical stimulation can improve motor recovery by ameliorating use-dependent plasticity impairment after stroke. Moreover, stroke rehabilitation strategies such as mental practice, virtual reality, and mirror therapy have been developed based on multisensory feedback, which plays an important role in reestablishing the disrupted sensorimotor loop after stroke. The efficacy of each motor neurorehabilitation technique has been validated, however the additive effects of combined neurorehabilitation therapies remain to be elucidated. As described above, when different stroke neurorehabilitation therapies are combined, the timing of these programs must be considered in the context of Hebbian plasticity and homeostatic metaplasticity. In this section, we discuss these combined neurorehabilitation therapies in two sections describing different and simultaneous timing strategies, respectively.

### Different timing between neurorehabilitation strategies

The concept of priming has helped us to understand the interaction between different neurorehabilitation processes when interventions are performed separately. Behavioral, environmental, pharmacological, or electrophysiological stimulation have been utilized as priming strategies that modify the effects of a subsequent intervention. In particular, many studies have utilized NIBS, such as repetitive TMS (rTMS) and tDCS, to prime cortical stimulation because there is strong evidence that this can change cortical excitability (Pascual-Leone et al., 1994; Chen et al., 1997; Nitsche and Paulus, 2000 and for review, see Lefaucheur, 2009). At present, increasing the excitability of the ipsilesional M1 by NIBS has been extensively studied to enhance use-dependent plasticity induced by physical therapy (Hummel et al., 2005; Kim et al., 2006; Takeuchi et al., 2008; Di Lazzaro et al., 2010). The interhemispheric competition model after stroke suggests that the excitability of the ipsilesional M1 can be increased by direct excitatory NIBS over ipsilesional M1 or indirect inhibitory NIBS over the contralesional M1 *via* a reduction in excessive interhemispheric inhibition from the contralesional to ipsilesional M1 (Hummel et al., 2005; Takeuchi et al., 2005; Kim et al., 2006 and for reviews, see Hummel and Cohen, 2006; Takeuchi and Izumi, 2012b).

A previous study has shown the importance of using priming NIBS for motor training by administering either inhibitory 1 Hz or sham rTMS over the contralesional M1 immediately before or after physical therapy (Avenanti et al., 2012). rTMS improved motor function in the paretic hand, regardless of whether it was administered before or after physical therapy; however, the group receiving rTMS before physical therapy showed robust and stable improvements when compared with the group receiving rTMS after physical therapy. In addition, bilateral priming stimulation improves the effect of physical therapy in stroke patients. Takeuchi et al. investigated whether motor training after bilateral rTMS improves motor learning in the paretic hand of patients with chronic stroke (Takeuchi et al., 2009). They found that bilateral rTMS, which consisted of alternating 1 and 10 Hz rTMS over the contralesional and ipsilesional M1, respectively, improved the effect of subsequent motor training more than unilateral stimulation. Sung et al. have shown that a combined protocol of inhibitory rTMS over the contralesional M1 followed by excitatory iTBS over the ipsilesional M1 enhances the effect of the conventional physical rehabilitation when compared with that of unilateral stimulation alone (Sung et al., 2013).

However, it is noted that bilateral stimulation may reduce synergic effects due to homeostatic metaplasticity. Ragert et al. reported that bilateral stimulation obeys homeostatic mechanisms operating across hemispheric boundaries and regulates the excitability of M1 in a manner similar to that seen for unilateral stimulation in healthy subjects (Ragert et al., 2009). They showed that the application of either inhibitory 1 Hz rTMS over the right M1 or excitatory iTBS over the left M1 results in increased cortical excitability in the left M1 relative to sham interventions. Preconditioning with 1 Hz rTMS over the right M1 significantly attenuated the excitability-enhancing effect of subsequent iTBS over the left M1, in line with homeostatic metaplasticity. Therefore, the positive results indicating that bilateral stimulation in stroke patients had a synergic effect on motor recovery seem to operate through different mechanisms than conventional homeostatic metaplasticity in healthy subjects. A recent study that the order of bilateral hemisphere stimulation was important for synergic effects might suggest that bilateral stimulation in stroke patients is less influenced by homeostatic metaplasticity. Wang et al. performed either a priming protocol with inhibitory 1 Hz rTMS to the contralesional M1 and subsequent excitatory iTBS to the ipsilesional M1 or the reverse, followed by conventional rehabilitation in stroke patients (Wang et al., 2014a). The first combination induced a better improvement in hand function than the second combination. As a possible mechanism, inhibitory rTMS over the contralesional M1 in patients with stroke could not fully enhance the excitability of the ipsilesional M1, in contrast to the findings for healthy subjects; therefore, homeostatic metaplasticity might not occur with bilateral stimulation.

In addition to physical therapy, priming NIBS facilitates the effect of other stroke motor neurorehabilitation methods, such as robot training, virtual reality, peripheral sensorimotor stimulation, and CIMT (Table 1). As another form of priming, many studies have investigated whether pharmacological intervention improves motor learning in stroke patients. Clinical trials suggest that pharmacological interventions, such as dextroamphetamine (Schuster et al., 2011), selective serotonin reuptake inhibitors (Chollet et al., 2011), donepezil (Berthier et al., 2003), or levodopa (Rosser et al., 2008) may facilitate physical therapy. Pharmacological intervention is also expected to enhance the effect of stroke neurorehabilitation for motor recovery (Nadeau et al., 2004; Wang et al., 2014b). Furthermore, although this finding is slightly different from the concept of priming, several studies have reported that a combination of stroke neurorehabilitation therapies based on distinct mechanisms can improve motor function in stroke patients (Page et al., 2009; Sun et al., 2010; Hsieh et al., 2014; Yoon et al., 2014) (Table 1).

**Table 1:** **Summary of studies using different timing between neurorehabilitation therapies**.

**Authors**	**Study design**	**Stroke phase**	**Priming/subsequent intervention**	**Control**	**Combined regime**	**Combination session**	**Behavioral results**
Edwards et al., 2009	Uncontrolled study	Chronic	Anodal tDCS/RT *n* = 6	None	1 h RT after 20 min tDCS over ipsilesional M1	Once	Not explored
Koganemaru et al., 2010	Cross-over	Chronic	NMES/5 Hz rTMS *n* = 9	NMES/Sham rTMS *n* = 9 5 Hz rTMS at rest *n* = 9	8 s rTMS over ipsilesional M1 after 50 s, 1 Hz paretic wrist and fingers extension aided by NMES	15 cycles (1 day)	rTMS/NMES improved grip power more than rTMS or NMES alone
Kim et al., 2014a	Cross-over	Subacute	Anodal tDCS/VR *n* = 15	tDCS alone *n* = 15 VR alone *n* = 15	15 min VR after 20 min tDCS over ipsilesional M1	Once	Not explored
Yamada et al., 2014	Open-label pseudo-RCT	Chronic	BTX/1 Hz rTMS plus intensive CR *n* = 42	1 Hz rTMS plus intensive CR *n* = 38	rTMS over contralesional M1 followed by intensive CR on the next day, after BTX injection	12 times (2 weeks)	rTMS/CR after BTX improved UEFM more than rTMS/CR
Page et al., 2009	Single-blind RCT	Chronic	CIMT/MP *n* = 5	CIMT alone *n* = 5	30 min MP after CIMT (3 days/weeks) and CIMT alone (2 days/weeks)	30 times (10 weeks)	CIMT/MP improved ARAT and UEFM more than CIMT alone
Sun et al., 2010	Single-blind RCT	Chronic	BTX/CIMT *n* = 16	BTX/CR *n* = 16	CIMT on the next day after BTX injection on paretic upper limb	36 times (3 months)	BTX/CIMT improved ARAT more than BTX followed by CR
Hsieh et al., 2014	Single-blind RCT	Chronic	2 weeks RT/2 weeks CIMT *n* = 16	4 weeks RT *n* = 16 4 weeks CR *n* = 16	2 weeks CIMT after 2 weeks RT	20 times (4 weeks)	RT/CIMT improved UEFM and WMFT more than RT or CR
Yoon et al., 2014	Single-blindRCT	Subacute	CIMT/MT *n* = 8	CIMT *n* = 9 CR *n* = 9	30 min MT after CIMT	10 times (2 weeks)	CIMT/MT improved BBT, Pegboard test, and grip power more than CIMT or CR
Pennati et al., 2014	Single-blind RCT	Chronic	BTX/RT *n* = 7	RT *r* = 8	RT few days after BTX injection on paretic upper limb	10 times (4 or 5 weeks)	BTX/RT and RT improved UEFM; no difference was observed between groups
Nadeau et al., 2004	Double-blind RCT	Chronic	Donepezil/CIMT *n* = 11	Placebo/CIMT *n* = 9	2 weeks CIMT during last period of 4 weeks taking donepezil (5 mg per day)	2 weeks	Donepezil/CIMT showed a tendency to improve WMFT, but there was no significant difference from placebo/CIMT
Malcolm et al., 2007	Double-blind RCT	Chronic	20 Hz rTMS/CIMT *n* = 9	Sham rTMS/CIMT *n* = 10	CIMT after 25 min rTMS over ipsilesional M1	10 times (2 weeks)	No difference was observed between groups
Theilig et al., 2011	Double-blind RCT	Subacute Chronic	1 Hz rTMS/NMES *n* = 12	Sham rTMS/NMES *n* = 12	20 min NMES on paretic wrist and finger extensors triggered by muscle activity, after 15 min rTMS over contralesional M1	10 times (2 weeks)	Both groups improved WMFT, but no difference was observed between groups
Wang et al., 2014b	Double-blind RCT	Subacute	Methylphenidate/Bilateral tDCS *n* = 3	Methylphenidate/Sham tDCS *n* = 3 Placebo/Bilateral tDCS *n* = 3	20 min tDCS was applied 1 h after drug intake (20 mg)	Once	Methylphenidate/bilateral tDCS improved Purdue pegboard score more than tDCS or drug alone
Gillick et al., 2015	Double-blind RCT	Congenital hemiparesis^*^	6 Hz primed 1 Hz rTMS/CIMT *n* = 10	Sham rTMS/CIMT *n* = 9	Real rTMS over contralesional M1 and CIMT are applied alternately every weekday	2 weeks	Not explored

*tDCS, transcranial direct current stimulation; RT, robot training; M1, primary motor cortex; NMES, neuromuscular electrical stimulation; rTMS, repetitive transcranial magnetic stimulation; VR, virtual reality; RCT, randomized controlled trial; BTX, botulinum toxin; CR, conventional rehabilitation; UEFM, upper extremity Fugl-Meyer score; CIMT, constraint-induced movement therapy; MP, mental practice; ARAT; action research arm test; WMFT, Wolf motor function test; MT, mirror therapy; BBT, box and block test. asterisk indicates that this study include young subjects (8–17 years)*.

However, it should be noted that a few studies have reported no synergic effect in combining priming intervention with stroke neurorehabilitation (Malcolm et al., 2007; Theilig et al., 2011; Pennati et al., 2014). Moreover, it remains to be evaluated whether combined neurorehabilitation provides the most optimal approach for motor recovery. As described above, combining neurorehabilitation therapies that target different mechanisms and have shorter intervals might be desirable for avoiding homeostatic metaplasticity and creating a synergic effect. Future study must clarify these questions.

### Simultaneous timing between neurorehabilitation therapies

Unlike combined neurorehabilitation strategies employing different timing, simultaneous interventions may provide more closed-loop strategies that target Hebbian plasticity. In this section, we discuss simultaneous timing between neurorehabilitation therapies, assessing muscle activity-dependent stimulation, brain state-dependent stimulation, and simultaneous combination neurorehabilitation therapies.

#### Muscle activity-dependent stimulation

Several studies have reported electromyography (EMG)-triggered neuromuscular electrical stimulation to induce motor recovery after stroke as combined sensorimotor stimulation synchronizes with muscle activity (Meilink et al., 2008; Shin et al., 2008; Fujiwara et al., 2009; Theilig et al., 2011). In addition, brain stimulation applied concurrently with voluntary movements is a form of muscle activity-dependent stimulation. Izumi et al. reported that muscle activity-dependent cortical stimulation synchronizes with hand movements in chronic stroke patients (Izumi et al., 2008). Nine patients were assigned to receive 100 pulses of active or sham 0.1 Hz TMS to the ipsilesional M1 during maximal effort paretic thumb and finger extension. Active TMS synchronized with maximum effort to induce a target movement that improved the motor function of the paretic hand when compared with sham TMS. Buetefisch et al. studied activity-dependent TMS combined with robot-assisted motor training in six chronic stroke patients (Buetefisch et al., 2011). Patients executed robot-assisted wrist extension movements at 0.2 Hz frequency while subthreshold 0.1 Hz TMS triggered by muscle activity was applied to the ipsilesional M1. This stimulation produced different map reorganization representing the muscle in the training movement when compared with robot-assisted motor training alone. However, this study did not show any difference in motor function between TMS synchronized with robot training and robot training alone. Massie et al. investigated muscle activity-dependent cortical stimulation synchronized with paretic hand movement using rTMS in patients with chronic stroke (Massie et al., 2013). Eighteen patients were assigned to receive 10 Hz rTMS during voluntary movement or 10 Hz rTMS at rest. Patients in the rTMS during movement group had 30 × 3 s 10 Hz rTMS over the ipsilesional M1 synchronized with lateral pinch contraction of the paretic hand. Activity-dependent rTMS with movement increased the cortical excitability of the ipsilesional M1 when compared with rTMS delivered during rest. However, there was no difference in pinch force between the two groups. At present, there is little evidence of muscle activity-dependent cortical stimulation in stroke patients. Moreover, the strict timing for LTP-like plasticity should be considered because brain stimulation that is delayed from movement onset reduces the excitability of the motor cortex in healthy subjects (Thabit et al., 2010; Massie et al., 2015).

#### Brain state-dependent stimulation

Muscle activity-dependent stimulation is not applicable for the stroke patients with severe hemiparesis who have no voluntary muscle activation. Therefore, these patients require activity-dependent stimulation paradigms based on brain activity. This concept is called brain state-dependent stimulation and has been developed in mainly animal studies using the action potentials of single neurons from an implanted electrode (Jackson et al., 2006; Rebesco et al., 2010). Recently, brain state-dependent stimulation has been investigated in humans *via* the development of brain-computer interface (BCI) techniques that can monitor brain activity in real time. Brain state-dependent stimulation using a BCI typically utilizes robot-assisted movement execution that is synchronized with brain activity during voluntary movement intention. It is postulated that the re-establishment of the disrupted sensorimotor loop by integrating movement intention and passive limb movement, assisted by a robot, will strengthen the associative connection, following the principles of Hebbian plasticity. Ramos et al. investigated whether robotic orthosis feedback using an electroencephalograph (EEG)-based BCI could improve physical therapy (Ramos-Murguialday et al., 2013). Thirty chronic stroke patients with severe hand weakness were randomly assigned to either the relevant or irrelevant feedback group. The results showed that the relevant online orthosis feedback improved subsequent physical therapy in chronic stroke patients with severe hemiparesis when compared with the random irrelevant feedback group.

Ang et al. has investigated whether an EEG-based motor imagery (MI) BCI coupled with robot-assisted hand grasping has an additional effect on therapist-assisted arm mobilization (Ang et al., 2014b). Twenty-one patients with chronic stroke were randomly allocated to the MI BCI coupled with robot feedback (BCI-robot training), robot training, or standard arm therapy group. Eighteen sessions of BCI-robot training led to additional improvement in therapist-assisted arm mobilization when compared with standard arm therapy; however, there was no significant difference between BCI-robot training and robot training alone. Another study by this group reported that sensorimotor feedback using a BCI had no additional effect on robot training-based neurorehabilitation (Ang et al., 2014a). Thus, using brain state-dependent activity stimulation as a neurorehabilitation therapy is very attractive, but still in its infancy; therefore, the optimal timing between brain activity and haptic feedback for Hebbian plasticity is yet to be elucidated.

#### Simultaneous combination neurorehabilitation therapies

tDCS has an advantage in that it can be simultaneously performed with other interventions, as it is not limited by movement. Therefore, many researchers have explored simultaneous tDCS and physical therapy to improve motor function in stroke patients (Lindenberg et al., 2012; Stagg et al., 2012; Zimerman et al., 2012). As with priming strategies, it has been reported that bilateral stimulation using tDCS during physical therapy also improved motor function in patients with stroke (Lindenberg et al., 2010b, 2012). tDCS during motor training might avoid a reduction in synergic effects due to homeostatic metaplasticity in patients with stroke as well as healthy subjects (Nitsche et al., 2003; Stagg et al., 2011). However, it remains to be evaluated whether tDCS before or during motor training is optimal for motor recovery, because at the present there is no evidence that simultaneous tDCS and motor training is more effective for motor recovery than priming tDCS. Moreover, to our knowledge, there are no reports describing activity-dependent tDCS in which short duration tDCS is repeatedly paired with volitional activity in order to induce Hebbian plasticity in a strict temporal relationship.

In addition to physical therapy, several studies have reported that the use of tDCS during neurorehabilitation procedures such as peripheral nerve stimulation, robot-assisted arm training, virtual reality training, and CIMT, facilitates motor recovery after stroke (Celnik et al., 2009; Bolognini et al., 2011; Ochi et al., 2013; Lee and Chun, 2014) (Table 2). As with different timing strategies, other simultaneous combined stroke neurorehabilitation therapies have been investigated to improve motor recovery (Reinkensmeyer et al., 2012; Mihara et al., 2013; Kim and Lee, 2015; Lin et al., 2014) (Table 2). Considering concepts of simultaneous timing, multisensory stimulation strategies such as motor imagery, mirror therapy, and virtual reality might be easy to combine with neurorehabilitation based on motor training. However, it remains to be evaluated which combined neurorehabilitation approaches are effective for motor recovery. Moreover, simultaneous combinations of neurorehabilitation approaches are not always synergistically effective for motor recovery in stroke patients. Hesse et al. studied the use of simultaneous tDCS and robot-assisted arm training in subacute stroke patients with severe motor function deficits and found that neither inhibitory tDCS over the contralesional M1 nor excitatory tDCS over the ipsilesional M1 enhanced the effect of robot-assisted arm training when compared with robot-assisted training alone (Hesse et al., 2011). Although, this study included patients with severe motor deficit, it should be considered that homeostatic plasticity might reduce synergic effects even in the case of simultaneous combinations. In line with this, Nitsche et al. suggested that simultaneous excitatory tDCS and LTP-like plasticity induced by PAS reduces corticospinal excitability in healthy subjects (Nitsche et al., 2007).

**Table 2:** **Summary of studies applying simultaneous neurorehabilitation therapies**.

**Authors**	**Study design**	**Stroke phase**	**One/other intervention**	**Control**	**Combined regime**	**Combination session**	**Behavioral results**
Hesse et al., 2007	Uncontrolled study	Subacute	Anodal tDCS/RT *n* = 10	None	7 min tDCS over ipsilesional M1 was applied at beginning of 20 min RT	30 times (6 weeks)	Three patients showed improved UEFM, but change in UEFM was small in seven patients with cortical lesion
Koyama et al., 2014	Uncontrolled study	Chronic	1 Hz rTMS/NMES *n* = 15	None	Onset of rTMS over contralesional M1 and NMSE on paretic wrist extensor (0.5 s on and 0.5 s off) were synchronous	24 times (2 weeks) twice a day	rTMS/NMSE improved UEFM, WMFT, and BBT
Celnik et al., 2009	Cross-over	Chronic	Anodal tDCS/PNS *n* = 9	Sham tDCS/PNS *n* = 9 tDCS/sham PNS *n* = 9	20 min tDCS at the end of 2 h PNS of median and ulnar nerve on paretic side	Once	tDCS/PNS improved motor learning more than tDCS or PNS
Ochi et al., 2013	Cross-over	Chronic	Anodal tDCS/RT *n* = 18	Cathodal tDCS/RT *n* = 18	10 min anodal (catodal) tDCS over ipsilesional (contralesional) M1 was applied at beginning of RT	5 times (1 week)	Both tDCS/RT improved UEFM, but no difference was observed between anodal and cathodal
Geroin et al., 2011	Single-blind RCT	Chronic	Anodal tDCS/RT gait *n* = 10	sham tDCS/RT gait *n* = 10 CR (gait) *n* = 10	7 min tDCS over ipsilesional M1 was at the start of 20 min robot-assisted gait training	10 times (2 weeks)	tDCS/RT and RT alone improved 6 min and 10 m walking more than CR. However, no difference was observed between tDCS/RT and RT alone
Reinkensmeyer et al., 2012	Single-blind RCT	Chronic	RT/VR *n* = 13	CR *n* = 13	1 h RT in VR environmental	24 times (2 months)	RT/VR improved UEFM, BBT, and grip power more than CR
Lee and Chun, 2014	Single-blind RCT	Subacute	Cathodal tDCS/VR *n* = 19	Cathodal tDCS/CR *n* = 20 VR alone *n* = 20	20 min tDCS during VR	15 times (3 weeks)	tDCS/VR improved MFT and UEFM more than tDCS/CR or VR
Kim and Lee, 2015	Single-blind RCT	Chronic	NMES/MT *n* = 10	Non-synchronized NMES/MT *n* = 10 CR *n* = 9	30 min NMES on paretic wrist extnsor, triggerd by muscle activity of non-paretic side during MT	20 times (4 weeks)	NMES/MT improved JTHT and BBT more than non-synchronized NMES/MT or CR
Kim et al., 2014b	Single-blind RCT	Subacute	NMES/MT *n* = 12	NMES alone *n* = 11	30 min NMSE on paretic wrist and finger extensors during MT	20 times (4 weeks)	NMES/MT improved UEFM more than NMES alone
Lin et al., 2014	Single-blind RCT	Chronic	NMES/MT *n* = 8	MT alone *n* = 8	NMES on paretic hand during 1 h MT	20 times (4 weeks)	NMES/MT improved BBT and ARAT more than MT alone
Ang et al., 2014a	Single-blind RCT	Chronic	MI/RT feedback *n* = 11	RT alone *n* = 14	MI synchronized with RT feedback of MI using EEG-based BCI	12 times (4 weeks)	MI/RT and RT improved UEFM, but no difference was observed between groups
Ang et al., 2014b	Single-blind RCT	Chronic	MI/RT feedback *n* = 6	RT alone *n* = 8 CR alone *n* = 7	MI synchronized with RT feedback of MI using EEG-based BCI	18 times (6 weeks)	MI/RT feedback and RT improved UEFM more than CR, but no difference between MI/RT feedback and RT
Lindenberg et al., 2010b	Double-blind RCT	Chronic	Bilateral tDCS/CR *n* = 10	Sham tDCS/CR *n* = 10	30 min tDCS was applied at beginning of 60 min CR	5 times	Bilateral tDCS/CR improved UEFM and WMFT more than sham tDCS/CR
Bolognini et al., 2011	Double-blind RCT	Chronic	Bilateral tDCS/CIMT *n* = 7	Sham tDCS/CIMT *n* = 7	40 min tDCS was applied at beginning of CIMT	10 times (2 weeks)	Bilateral tDCS/CIMT improved JTHF and UEFM more than sham tDCS/CIMT
Hesse et al., 2011	Double-blind RCT	Subacute	Aodal tDCS/RT *n* = 32	Sham tDCS/RT *n* = 32 Cathodal tDCS/RT *n* = 32	20 min tDCS during RT	30 times (6 weeks)	All patients showed UEFM improvement, but no difference was observed between groups
Mihara et al., 2013	Double-blind RCT	Chronic	MP/Visual feedback *n* = 10	MP/Irrelevant visual feedback *n* = 10	10 min MP with visual feedback of MI using NIRS-based BCI	6 times (2 weeks)	MP/Visual feedback improved UEFM more than MP/irrelevant feedback
Ramos-Murguialday et al., 2013	Double-blind RCT	Chronic	MI/Relevant RT feedback *n* = 16	MI/Irrelevant RT feedback *n* = 14	MI with relevant RT feedback of MI using EEG-based BCI	20 times (4 weeks)	MI/rerevant RT feedback improved UEFM more than MI/irrelevant RT feedback

*tDCS, transcranial direct current stimulation; RT, robot training; M1, primary motor cortex; UEFM, upper extremity Fugl-Meyer score; rTMS, repetitive transcranial magnetic stimulation; NMES, neuromuscular electrical stimulation; WMFT, Wolf motor function test; BBT, box and block test; PNS, peripheral nerve stimulation; RCT, randomized controlled trial; CR, conventional rehabilitation; VR, virtual reality; MFT, manual function test; MT, mirror therapy; JTHF, Jebsen Taylor hand function Test; ARAT; action research arm test; MI, motor imagery; EEG, electroencephalography; CIMT, constraint-induced movement therapy; MP, mental practice; NIRS, near-infrared spectroscopy*.

Thus, many studies have reported positive synergic effects of combined neurorehabilitation therapies in both different and simultaneous timing strategies; however, the outcome of these combination-based strategies is influenced by several factors. At the present, there have been many reports of the use of neurorehabilitation combinations that include NIBS. NIBS combination strategies use the interhemispheric competition model after stroke as rationale; however, applying this theory to all patients with stroke is oversimplified or even incorrect (Di Pino et al., 2014). In fact, several studies have reported that NIBS might have no effect of motor recovery in some stroke patients with acute phase or cortical lesions (Ameli et al., 2009; Seniow et al., 2012; Rossi et al., 2013). Moreover, in contrast to the usual NIBS strategies based on the interhemispheric competition model, an exploratory study has reported that priming with an inhibitory continuous TBS (cTBS) over the ipsilesional M1 improved subsequent motor training in the paretic upper extremity through homeostatic metaplasticity (Di Lazzaro et al., 2013). These results indicate that the effect of combination therapies depends not only on the timing, type and intensity of intervention, but also phase and lesion site after stroke, possibly due to heterogeneous neural reorganization responses to stroke. Similarly, homeostatic metaplasticity and Hebbian plasticity might vary according to stroke state. In future research, neurophysiological and functional neuroimaging studies must elucidate the mechanisms of heterogeneous responses to combined therapies in patients with stroke. In addition to clinical measurement, such as severity of motor function, the evaluation of corticospinal tract integrity and lesion size/location using neurophysiological and neuroimaging techniques also may be useful to control for variability of stroke lesions (Stinear et al., 2007; Lindenberg et al., 2010a; Zhu et al., 2010). In next section, we discuss methods for facilitating combination therapy in the context of homeostatic metaplasticity and Hebbian plasticity.

## Facilitating the synergic effect of combined neurorehabilitation therapies

The synergic effect of combined neurorehabilitation therapies will vary according to the type and timing of the neurorehabilitation used. To facilitate this synergic effect, it is important to predict its effect by confirming the mechanism involved, in addition to improving new technologies. We have mainly discussed peripheral sensorimotor feedback as a brain-state dependent stimulation, but simultaneous cortical and peripheral stimulation using BCI technologies could provide a novel neurorehabilitation strategy for stroke patients. Preliminary work decoding brain signals of motor imagery has shown that synchronous TMS with haptic feedback assisted by a robot facilitated the induction of associative plasticity (Gharabaghi et al., 2014). In addition to synchronous cortical and peripheral stimulation, real-time monitoring of brain activity will reveal the optimal timing between different neurorehabilitation therapies. The development of technology that decodes brain activity might facilitate the synergic effect of combined stroke neurorehabilitation by monitoring the intrinsic variations in neuronal excitability, which might influence homeostatic metaplasticity and Hebbian plasticity.

The synergic effect might be facilitated when it is combined with multisensory simulation, such as motor imagery, action observation, mental practice, mirror therapy, training in virtual reality, and music-related therapies (Johansson, 2012). Multisensory training protocols better approximate natural settings and are more effective for learning (Shams and Seitz, 2008; Johansson, 2011). Moreover, multisensory stimulation strategies induce activation of higher order association areas as well as unimodal sensory areas (Ghazanfar and Schroeder, 2006; Johansson, 2012). Therefore, multiple regions activated by multisensory stimulation might have associative connections with the motor cortex, resulting in enhanced Hebbian plasticity. Moreover, combination neurorehabilitation strategies based on motor learning and multisensory stimulation might avoid a convergence of LTP-like plasticity on M1 that reduces the synergic effect via homeostatic metaplasticity. Furthermore, the effects of emotional motivation that can be activated by multisensory stimulation, such as music and virtual reality interactive games, could engage reward–learning networks, helping to facilitate the motor recovery (Camara et al., 2009; Rodriguez-Fornells et al., 2012; Novak et al., 2014).

If associative plasticity between multiple regions and M1 is important for the synergic effect of combined neurorehabilitation, direct brain stimulation outside M1 may also enhance the neurorehabilitation. Although we have primarily discussed M1 as the brain stimulation site for neurorehabilitation, several studies have shown neuroplastic changes induced by stimulating other areas of the brain that are functionally connected to M1, such as the somatosensory (Bliem et al., 2008), supplementary (Hamada et al., 2009), and premotor (Potter-Nerger et al., 2009) cortices, and the cerebellum (Popa et al., 2013). A previous study has revealed that inhibitory cTBS to both the contralesional M1 and primary somatosensory cortex can enhance motor improvement owed to practice with the paretic hand in patients with chronic stroke when compared with sham stimulation (Meehan et al., 2011). Moreover, the parameters of improvements in motor function were different between stimulation over M1 and the somatosensory cortex, indicating that stimulation beyond M1 may have a differential impact on stroke rehabilitation. Activation of movement-related components such as sensation, proprioception, motor planning, and coordination might enhance motor recovery by new associative plasticity pathways beyond M1.

In this regard, functional and structural connectivity neuroimaging information will be helpful in better understanding the heterogeneity of associative plasticity and to allow the individual design of combined neurorehabilitation programs based on preserved and undamaged brain connectivity (Rehme et al., 2011; Grefkes and Ward, 2014). Moreover, the response to NIBS might reveal how homeostatic plasticity influences the synergic effect of combined neurorehabilitation therapies in patients with stroke. Several studies using NIBS have provided evidence regarding homeostatic metaplasticity (Lang et al., 2004; Fricke et al., 2011; Murakami et al., 2012). Many studies have assessed the combination of facilitatory techniques in patients with stroke; however, these approaches risk degradation of subsequent learning according to homeostatic metaplasticity. The threshold and degree of homeostatic metaplasticity itself might change after stroke, which may contribute to the mixed results seen with combined neurorehabilitation therapies. Therefore, NIBS programs could serve as diagnostic tools of responsiveness to combined neurorehabilitation therapies by evaluating the threshold and degree of homeostatic metaplasticity in individual patients with stroke.

Plasticity outside the brain is of interest in future studies using combined neurorehabilitation, as plasticity may occur in the spinal cord (Taylor and Martin, 2009). In particular, it is noted that the reorganization of spinal cord can influence the synergic effect of combined neurorehabilitation on gait disturbance (Geroin et al., 2011), because walking ability is related to both cortical and spinal levels of organization (central pattern generator). There is little evidence to date regarding plasticity in the spinal cord following neurorehabilitation (Motta-Oishi et al., 2013; Zheng et al., 2013); however, structural plasticity is known to occur after stroke in animal studies (for review, see Starkey and Schwab, 2014).

## Conclusion

Although meta-analysis and systematic reviews have investigated these therapies, there is a lack of convincing evidence to support any motor stroke neurorehabilitation approach as being more effective in recovery than any other approach (Langhorne et al., 2009; Hsu et al., 2012; Pollock et al., 2014; Veerbeek et al., 2014). Moreover, the effectiveness of motor neurorehabilitation varies across patients with stroke. Therefore, these techniques are being investigated to determine whether their combination has stable and synergistic effects in motor recovery. Nevertheless, combination techniques have shown mixed results. There is heterogeneity in the combined neurorehabilitation protocols that are used, for example, with respect to factors such as the number of sessions, intensity, type and timing of interventions, time after stroke, lesion site, severity of motor function, gender, pathology, and genetic factors, which adds complexity to this field. The change in the threshold and degree of Hebbian plasticity and/or homeostatic metaplasticity after stroke might influence the uncertainty of the synergic effect of combined neurorehabilitation. Few possible combinations of stroke neurorehabilitation have been tested experimentally; therefore, utilizing the concept of Hebbian plasticity and homeostatic metaplasticity can help in developing an appropriate combination of therapies for motor recovery in future studies.

### Conflict of interest statement

The authors declare that the research was conducted in the absence of any commercial or financial relationships that could be construed as a potential conflict of interest.
